# Winter Diet for Infants and Children

**Published:** 1906-12

**Authors:** Marion McH. Hull

**Affiliations:** Atlanta; Visiting Physician to the Presbyterian Hospital and to the Sheltering Arms; Professor of Materia Medica and Therapeutics in the Atlanta School of Medicine


ATLANTA

Journal-Record of Medicine

Successor to Atlanta Medical and Surgical Journal, Established 1855, and Southern Medical Record, Established 1870.

OWNED BY THE ATLANTA MEDICAL JOURNAL CO.

Published Monthly.

*Official Organ Fulton County Medical Society, State Examining Board, Presbyterian Hospital, Etc.*

VOL. VIII. DECEMBER, 1906. No. 9.

BERNARD WOLFF, M.D., EDITOR, 319-320 Prudential Bldg.

M. B. HUTCHINS, M.D., BUSINESS MANAGER, 1007-1008 Century Bldg.

E. G. BALLENGER, M.D., ASSOCIATE EDITOR AND ASSISTANT MANAGER.

### *ORIGINAL COMMUNICATIONS.*

### WINTER DIET FOR INFANTS AND CHILDREN.*

BY MARION McH. HULL, M.Sc., M.D.,

Visiting Physician to the Presbyterian Hospital and to the Sheltering Arms; Professor of Materia Medica and Therapeutics in the Atlanta School of Medicine.

The dietetic treatment of infants and children, in fact of adults also, is so important that it would not take long to make a choice between the physician who paid a great deal of attention to diet and neglected the medicinal treatment and the one who paid a great deal of attention to the medicinal and neglected the dietetic. But there is no necessity for any physician to neglect either. The progressive, conscientious physician of today no longer dismisses the subject of diet with general instructions to the mother to "be careful of the diet" any more than he would tell her to be careful what drugs she gave her

* Read before the Fifth District Medical Society at Atlanta, Ga, October 18, 1906.

child. Success often depends upon the same preciseness in selecting the articles of diet as in prescribing other remedies.

This is also a day of preventive medicine; we are striving to do all in our power to develop a patient's resistance, so that he will not succumb to disease; in this greatest work of the modern physician, diet is *par excellence* his most important yoke-fellow.

As diet differs with different ages and climates, so it does with different seasons. The inhabitant of the tropics lives mostly on fruits and vegetables; the Eskimo lives mostly on meats and fats. This is partly due to the supply being more readily obtained in these respective articles, but it is also because the different climates require different foods to meet the demands of the body. So the child in winter has different demands from those in summer, and a consideration of these we hope will be helpful.

The object of food is to build new tissue, to restore waste, and to maintain heat. We eat to live, not live to eat. A knowledge of three elements is essential to the solution of our problem. These are (a) the purposes of the various foodstuffs; (b) the demands of the child's body in winter and (c) the composition and digestibility of the various foods at our disposal.

The foodstuffs fall into five classes—water, salts, protein, carbohydrates, and fats. The first two are essential at all times in about the same quantities, except that more water is required in hot weather. The purpose of water is as a solvent and as a vehicle. The salts are appetizers, and are necessary to the repair of waste and building of new tissue. Of the remaining three the proteins are most essential, since from them are built the muscular, nervous and bony tissues, and the power of the body supplied. The carbohydrates and fats furnish heat and energy to the body, and are stored up as fat for protection and future use. All of these three are interchangeable—that is, any one may be made to do the work of all three, but only at great expense to the body metabolism. For instance, it would require six pounds of lean beef a day to furnish the amount of heat and energy that the ordinary amount of carbohydrates needed (about one pound) would do in a better manner, and this would soon tax the digestion to such an extent that the body would be unable to digest that amount of meat. Mixed in the proper proportions, therefore, the body gets the maximum results at minimum expenditure.

The second essential element—the demands of the child's body in winter—will be manifest on thought. There must be greater heat production to compensate for the greater amount lost through radiation; there must be more energy produced to compensate for the greater amount expended in the greater activity of the body in winter; and there must be more fat stored up to furnish protection to the body.

It is a common experience to find that we increase in weight in the cold months, in spite of the greater amount of exercise that we take. It is one of nature's ways of preserving energy, putting on a better covering of fat so that so much more energy will not have to be spent in heat production.

The third element of the problem is a knowledge of the composition and the caloric value of the different kinds of foods. We know from the former elements that to meet the demands a larger quantity of all of the foods is needed, and that more protein and fat are needed proportionately as well as actually. Therefore, a knowledge of the composition and caloric value of the foods at our disposal will enable us to know which will give the greatest amounts of protein and fats with the proper proportion of carbohydrates. These will manifestly be selected in choosing the proper winter diet.

In these considerations only infants over one and one-half years, who have been put on other than a milk diet, are taken into account, and due consideration must be made for their respective ages.

The following table, made after Frühwald, will show the composition and relative fuel values of certain available articles, and in the order of value:

| | Total percentage of nutrient matter. | Proteids. | C. & F. | Food value per pound. |
|---|---|---|---|---|
| Butter | 90 % | | 90 % | 3600 calories |
| Breakfast Bacon | 75 " | 15 % | 70 " | 3000 " |
| Oatmeal | 90 " | 15 " | 75 " | 1800 " |
| Sugar | 100 " | | 100 " | 1800 " |
| Rice | 95 " | 5 " | 90 " | 1700 " |
| Wheatbread | 90 " | 12 " | 78 " | 1600 " |
| Cornmeal | 85 " | 10 " | 75 " | 1600 " |
| Beans | 85 " | 20 " | 65 " | 1500 " |
| Beef (sirloin) | 40 " | 20 " | 20 " | 1200 " |
| Cream | 25 " | 4 " | 21 " | 1000 " |
| Eggs | 30 " | 15 " | 15 " | 800 " |
| Potatoes | 17 " | 2 " | 15 " | 400 " |

Lamb has more fat than beef, and beef than chicken, and the two first are better winter foods. Fish is rich in fat and is a good winter food. Of the cereals, beside oatmeal and cornmeal, buckwheat is rich in fat and proteids and is an excellent winter food. Macaroni is rich in proteids and carbohydrates and is a valuable food for winter use.

Butter is particularly useful. From one to two ounces a day may be given a child of three to five years. Melted butter should be prohibited, as in the process of heating the fat is broken up into its component glycerine base and fatty acids (pelmetic, stearic and oleic) and these latter are very difficult of digestion.

With this information at hand we are able to determine the proper diet for winter ; that is, the food that will give the maximum amount of heat and energy to meet the body's private demand for increased heat production and increased energy. Such a diet should consist of plenty of butter, oatmeal, cornbread or cornmeal mush, beans, rice and cream, with breakfast bacon, beef, eggs and milk. Fruits and the more digestible vegetables should be given to counteract any constipating tendency and to supply the antiscorbutic element of raw foods; and variety, which is not only the spice of life but an essential in keeping the child's appetite up to normal, should be dispensed as needed.

To bring more forcefully before the mind the point I wish to emphasize, I append two schedules for the winter and summer diets for a normal child of from three to five years old.

| WINTER. | SUMMER. |
| --- | --- |
| **Breakfast, 7:30, a. m.** | |
| Fruit. | Fruit. |
| Oatmeal and cream and sugar. | Wheat cereal. |
| Beefsteak, breakfast bacon, lamb chops or eggs. | Eggs or breakfast bacon, milk or cream. |
| Light buckwheat cakes, butter and syrup. | Beaten biscuit. |
| **Lunch, 11 a. m.** | |
| Milk or bread and butter. | Fruit or lemonade. |
| **Dinner, 2:30, p. m.** | |
| Soup. | Chicken. |
| Cornbread, beans. | Rice and tomatoes. |
| Rice, potatoes, butter. | Peas, beans, squash. |
| Roastbeef, lamb or fish. | Sherbet or fruits. |
| Spinach. | |
| Rice custard, gelatine. | |
| Blanc-mange. | |
| **Supper, 6:30.** | |
| Cooked fruits. | Cooked fruit. |
| Cornmeal mush. | Cereal and milk. |
| Bread and butter. | |

### SUMMARY.

1. In which there is greater heat production, greater nitrogenous waste, and greater energy expended and more fat stored up.

2. More food is needed and a relatively larger supply of protein and fats; in contrast to summer when more water and carbohydrates are needed.

3. To meet these demands those foods should constitute the main articles of diet which contain more fat than protein and yield more calories per pound, principal among which are butter, breakfast bacon, oatmeal, cornmeal and buckwheat.

---

WHEN a skiagraph shows a condition not recognizable at once as a definite lesion, it is important to make an x-ray picture of the corresponding part of the body on the other side. It may show that the condition is merely a symmetrical peculiarity, and not a pathological one.—*American Jour. of Surgery*.

